# Factors Associated With Surveillance Testing in Individuals With COVID-19 Symptoms During the Last Leg of the Pandemic: Multivariable Regression Analysis

**DOI:** 10.2196/52762

**Published:** 2024-07-18

**Authors:** Timothy Dotson, Brad Price, Brian Witrick, Sherri Davis, Emily Kemper, Stacey Whanger, Sally Hodder, Brian Hendricks

**Affiliations:** 1 West Virginia Clinical and Translational Sciences Institute, Morgantown, WV, United States; 2 Department of Management Information Systems, West Virginia University, Morgantown, WV, United States; 3 Department of Public Health Sciences, College of Behavioral, Social, and Health Sciences, Clemson University, Clemson, SC, United States; 4 American Diabetes Association, Arlington, VA, United States; 5 School of Medicine, West Virginia University, Morgantown, WV, United States; 6 Center for Rural and Community Health, West Virginia School of Osteopathic Medicine, Lewisburg, WV, United States

**Keywords:** COVID-19, testing, symptomatic, RADx, rural, health disparities, regression analysis, surveillance, SARS-CoV-2, United States, asymptomatic, survey, demographic, clinical, behavioral, logistic regression, bivariate map, child, children, youth, adolescent, adolescents, teen, teens, teenager, teenagers, public health, machine learning, mHealth, mobile health, digital health, cross-sectional study, physical health, mental health, Rapid Acceleration of Diagnostics

## Abstract

**Background:**

Rural underserved areas facing health disparities have unequal access to health resources. By the third and fourth waves of SARS-CoV-2 infections in the United States, COVID-19 testing had reduced, with more reliance on home testing, and those seeking testing were mostly symptomatic.

**Objective:**

This study identifies factors associated with COVID-19 testing among individuals who were symptomatic versus asymptomatic seen at a Rapid Acceleration of Diagnostics for Underserved Populations phase 2 (RADx-UP2) testing site in West Virginia.

**Methods:**

Demographic, clinical, and behavioral factors were collected via survey from tested individuals. Logistic regression was used to identify factors associated with the presence of individuals who were symptomatic seen at testing sites. Global tests for spatial autocorrelation were conducted to examine clustering in the proportion of symptomatic to total individuals tested by zip code. Bivariate maps were created to display geographic distributions between higher proportions of tested individuals who were symptomatic and social determinants of health.

**Results:**

Among predictors, the presence of a physical (adjusted odds ratio [aOR] 1.85, 95% CI 1.3-2.65) or mental (aOR 1.53, 95% CI 0.96-2.48) comorbid condition, challenges related to a place to stay/live (aOR 307.13, 95% CI 1.46-10,6372), no community socioeconomic distress (aOR 0.99, 95% CI 0.98-1.00), no challenges in getting needed medicine (aOR 0.01, 95% CI 0.00-0.82) or transportation (aOR 0.23, 95% CI 0.05-0.64), an interaction between community socioeconomic distress and not getting needed medicine (aOR 1.06, 95% CI 1.00-1.13), and having no community socioeconomic distress while not facing challenges related to a place to stay/live (aOR 0.93, 95% CI 0.87-0.99) were statistically associated with an individual being symptomatic at the first test visit.

**Conclusions:**

This study addresses critical limitations to the current COVID-19 testing literature, which almost exclusively uses population-level disease screening data to inform public health responses.

## Introduction

### Pandemic Spread

The SARS-CoV-2 pandemic has had several waves of infection driven by the introduction and spread of multiple variants. In the United States, the first case of COVID-19 appeared in January 2020 [1]. The Alpha variant, first introduced in December 2020, comprised the second wave of the COVID-19 pandemic, occurring at the same time as vaccination campaigns were being rolled out in many states [2]. This was a critical moment in the SARS-CoV-2 timeline, as Alpha was the first variant of concern with adapted mutations, increasing the risk of person-to-person transmission [3]. The pattern of spread and speed of the third and fourth waves involving Omicron variants (BA1-3 and BA4-5) were wider reaching and faster than previous variants [4]. Unfortunately, this was problematic with reduced testing efforts making it more difficult to monitor SARS-CoV-2 infections in populations [5].

### Health Disparities

Urban US areas experienced the greatest burden of cases early in the epidemic with West Virginia, a largely rural state, being the last state to identify a confirmed case of COVID-19 in late January 2020. This is problematic as 50% of rural residents are at high risk of serious illness and hospitalization if they contract SARS-CoV-2 [6]. In West Virginia, the Alpha variant cases peaked in April 2021, 4 months after its initial detection in the United States and after vaccination campaigns were already underway, with subsequent peaks in October 2021 for the Delta variant and February 2022 for the first Omicron peak. Due to unequal access to health resources, the impacts of the disease vary throughout the state, particularly in southwest West Virginia where there are already health disparities [7].

### Prior Work

By August 2020, testing in the United States had peaked at around 1 million tests per day, at which point COVID-19 had become the third leading cause of death [1]. COVID-19 testing data have been used in machine learning models and spatial epidemiological studies to help identify disparities in testing and outcomes for COVID-19, and guide public health policies [8-10]. Previous studies have analyzed socioeconomic status, race, comorbidities, mental health, and substance abuse effects to identify disparities and seasonal impacts [7,9-14]. Furthermore, other epidemic models have been tested using COVID-19 data to help forecast future SARS-CoV-2 waves and look at the impact of testing itself [11,12]. However, previous research was conducted during times with high-to-moderate community testing, when data more accurately reflected the general population’s risk of disease and allowed for standard epidemiological models of study [1,13-15]. The pandemic is now in a phase where those seeking testing are largely symptomatic. Lower testing and increasing reliance on home testing for COVID-19 have created a situation in which traditional epidemiological measures are suboptimal [16].

### Study Objectives

Subsequent waves of SARS-CoV-2 infection are now monitored with varying surveillance efforts, which have dwindled from the population to the community level as the focus shifted away from testing to preventing severe infection and health care strain [5]. This situation has resulted in a heightened reliance on testing data from people who are symptomatic and seek out testing. Currently, few studies have examined the demographics, clinical factors, or barriers to testing among people who are symptomatic and seeking COVID-19 testing [15,17]. As such, the objectives of this study were to identify factors that increased the prevalence of individuals who were symptomatic at testing locations and assess whether there was spatial autocorrelation among the rate of tested people who were symptomatic and their residential zip code. Spatial autocorrelation, or clustering of tested people who were symptomatic, was assessed to better understand if geographic differences in hospital or doctor referrals potentially biased the number of people who were symptomatic who visited testing locations [18,19]. The results address current literature gaps concerning which factors are associated with test seeking and have the potential to inform public health policy to ensure COVID-19 testing services remain available to vulnerable populations living in the rural United States.

## Methods

### Data Source and Management

This cross-sectional study utilized questionnaire data collected for phase 2 of the Rapid Acceleration of Diagnostics for Underserved Populations program (RADx-UP2). Detailed information on RADx-UP2, including program aims and research projects, has been described elsewhere [20]. Briefly, RADx-UP is a multisite National Institutes of Health–funded project, developed to disseminate testing resources within communities of varying social or economic vulnerability [21]. All sites are required to include common data elements (CDEs) in their data collection instruments to harmonize data across states [21]. Project CDEs include an individual’s address, demographics, clinical comorbidities, signs and symptoms at the time of testing, behavioral data, and more [21]. For the West Virginia site, data were collected using ArcGIS Survey 123 (Esri) at testing events. The benefits of using syndromic surveillance for public health programming and response have been described in previous public health research elsewhere [16,22-24].

### Ethical Considerations

Approval for this study was given by the West Virginia University Institutional Review Board (protocol 2202534378A001). Informed consent was requested on the survey used for data collection in the form of an opt-out question. All included data has been deidentified for analysis and publication. Site-level incentives were developed in conjunction with community partners that assisted in hosting testing events. As such, these incentives varied by site and community and consisted of monthly raffles for all who participated in a survey during that month for each testing site, with prizes including technology, tickets to sporting events, and outdoor items. Other incentives included t-shirts and plastic reusable cups for participation.

### Inclusion Criteria

The study inclusion criteria were any individual tested at a West Virginia RADx-UP2 testing site from May 2022 until November 2022. Testing sites included pharmacies, hospitals, and homeless shelters in underserved areas throughout the state manned by RADx-UP2 staff as well as at testing events such as Solutions Oriented Addiction Response (SOAR) meetings and other community-sponsored events. Individuals seeking COVID-19 testing paid for by RADx-UP funding and consented to have their information collected. For this study, the analytic sample was limited to information collected at each individual’s first test, including the individual’s demographics, signs and symptoms, history of chronic disease, receipt of two vaccine doses, and challenges or motivators to seek care. The study outcome was the odds of an individual who was symptomatic (vs asymptomatic) being seen at the time of first testing. Individuals who were symptomatic presented with one of the following at the time of testing: fever or chills, cough, shortness of breath or difficulty breathing, lack of energy or general tired feeling, muscle or body aches, headache, new loss of taste or smell, sore throat/congestion or runny nose, feeling sick to your stomach or vomiting/diarrhea, abdominal pain, or skin rash.

### Predictor Covariates

Predictor covariates in the analysis included age categories (<18, 19‐29, 30‐39, 40‐49, 50‐59, and ≥60 years), race, sex at birth, whether a person is an essential worker, whether a person is fully vaccinated (eg, received two doses of Moderna/Pfizer or one dose of Johnson & Johnson), presence/absence of physical or mental health conditions (yes/no), six challenges to health (yes/no), specific barriers to testing (yes/no), and a measure of economic distress based on the individual’s zip code of residence to adjust for nonrandom community-level effects. Physical health conditions, mental health conditions, and barriers to testing were combined into their groupings due to the small sample size of the individual and missingness in the subgroups. Physical health conditions included immunocompromised condition, autoimmune disease, hypertension, diabetes, chronic kidney disease, cancer diagnosis or treatment within the past 12 months, cardiovascular disease, asthma, chronic obstructive pulmonary disease, other chronic lung disease, and sickle cell anemia. Mental health conditions included depression, alcohol or substance use disorder, intravenous drug use, and other mental health disorders. The six challenges to health included access to mental/physical health care, having a place to stay/live, getting enough food to eat, having clean water to drink, getting the medicine needed, and having transportation from one place to another. Barriers to testing included protected time off to visit a testing site; out-of-pocket costs for test; out-of-pocket costs for transportation, childcare, or time off work to get tested; knowledge of where testing is done in their community; pain or discomfort from the test or saliva collection; and concern about others handling their personal data. All predictor covariates, except for the economic distress score, were collected as CDEs required for all funded RADx-UP2 projects [21]. The zip code–level Distressed Communities Index (DCI) was linked to survey data by individual zip code of residence to adjust for nonrandom selection of underserved communities for testing. The DCI is a measurement of community economic disparities that consists of seven measures obtained from the US Census Bureau’s American Community Survey: no high school diploma, housing vacancy rate, adults not working, poverty rate, median income ratio, changes in employment, and changes in establishments. This was critical as RADx-UP2 nonrandomly selects communities for testing based on whether they are underserved. DCI was a continuous variable, where higher numbers indicated more distress [25]. This index of socioeconomic deprivation has been utilized in previous social epidemiology literature to characterize health disparities [26,27].

### Statistical Analysis

Data were analyzed by multivariable logistic regression to evaluate the association between the odds of an individual being symptomatic at the time of the first test and each of the predictors. Interaction effects between the six challenges to health and the DCI were also included in the multivariable logistic regression model, and backward selection with Akaike information criterion was used to ensure the best covariates model was used adjusting for age, gender, and race after selection. Statistical significance was evaluated using adjusted odds ratios (aORs) and corresponding 95% CIs at an α level of .05. Tests for global spatial autocorrelation (clustering) of individuals who were symptomatic were conducted using a global Moran *I* value. Statistical spatial dependence was evaluated using the tests’ computed *z* score and *P* value [28]. All data management and regression analyses were conducted in R (The R Foundation for Statistical Computing). Spatial analysis and thematic maps displaying zip code–level relationships between the rate of people who were symptomatic per 10 individuals tested and the distressed communities score were created in ArcGIS Pro 2.9.2 (Esri).

## Results

### Data Source and Management

Of the 2103 testing questionnaires completed between May 7 and November 14, 2022, 1423 unique individuals were identified as having self-reported as being symptomatic at the time of their first test (Table 1). In the overall sample, 24.5% (n=348) were 60 years or older, 85.5% (n=1217) were White, and 51.7% (n=735) were female. The majority of individuals were vaccinated (n=975, 68.5%), did not report any physical (n=773, 54.3%) or mental (n=1120, 78.7%) health conditions, and did not have any of the six challenges to health: access to health care (n=1155, 81.2%), place to stay/live (n=1162, 81.7%), enough food to eat (n=1198, 84.2%), clean water to drink (n=1228, 86.3%), getting needed medication (n=1163, 81.7%), and having transportation (n=1165, 81.9%). Among individuals who were symptomatic, 26.6% (n=219) were 60 years or older, 87.1% (n=717) were White, and 55.8% (n=459) were female. Similar to the overall sample, the majority of individuals who were symptomatic were vaccinated (n=581, 70.6%), did not report any physical (n=418, 50.8%) or mental (n=656, 79.7%) health issues, and did not have any of the six challenges to health: access to health care (n=736, 89.4%), place to stay/live (n=746, 90.6%), enough food to eat (n=759, 92.2%), clean water to drink (n=773, 93.9%), getting needed medication (n=741, 90%), and having transportation (n=747, 90.8%).

**Table 1.** Demographics and clinical characteristics of individuals tested for SARS-CoV-2 during phase 2 of the Rapid Acceleration of Diagnostics for Underserved Populations program. The program took place between May 7 and November 14, 2022, and tested 1423 unique individuals who self-reported as being symptomatic at the time of their first test.

Variable			Individuals, n (%)	Clinical symptoms, n (%)	
			Nonsymptomatic (n=600)	Symptomatic (n=823)
**Age (years)**					
	≤18		285 (20.0)	110 (18.3)	175 (21.3)
	19-29		143 (10.0)	51 (8.5)	92 (11.2)
	30-39		208 (14.6)	100 (16.7)	108 (13.1)
	40-49		188 (13.2)	92 (15.3)	96 (11.7)
	50-59		164 (11.5)	74 (12.3)	90 (10.9)
	≥60		348 (24.5)	129 (21.5)	219 (26.6)
	Missing		87 (6.1)	44 (7.3)	43 (5.2)
**Race**					
	White		1217 (85.5)	500 (83.3)	717 (87.1)
	Black/African American		114 (8.0)	61 (10.2)	53 (6.4)
	Other		67 (4.7)	33 (5.5)	34 (4.1)
	Missing		25 (1.8)	6 (1.0)	19 (2.3)
**Sex at birth**					
	Female		735 (51.7)	276 (46.0)	459 (55.8)
	Male		651 (45.7)	307 (51.2)	344 (41.8)
	Missing		37 (2.6)	17 (2.8)	20 (2.4)
**Essential worker**					
	No		990 (69.6)	429 (71.5)	561 (68.2)
	Yes		319 (22.4)	118 (19.7)	201 (24.4)
	Missing		114 (8.0)	53 (8.8)	61 (7.4)
**Vaccinated**					
	No		392 (27.5)	182 (30.3)	210 (25.5)
	Yes		975 (68.5)	394 (65.7)	581 (70.6)
	Missing		56 (3.9)	24 (4.0)	32 (3.9)
**Physical health condition**					
	No		773 (54.3)	355 (59.2)	418 (50.8)
	Yes		472 (33.2)	168 (28.0)	304 (36.9)
	Missing		178 (12.5)	77 (12.8)	101 (12.3)
**Mental health condition**					
	No		1120 (78.7)	464 (77.3)	656 (79.7)
	Yes		233 (16.4)	110 (18.3)	123 (14.9)
	Missing		70 (4.9)	26 (4.3)	44 (5.3)
**Challenges to health**					
	**Access to health care**				
		No	1155 (81.2)	419 (69.8)	736 (89.4)
		Yes	232 (16.3)	173 (28.8)	59 (7.2)
		Missing	36 (2.5)	8 (1.3)	28 (3.4)
	**Place to stay/live**				
		No	1162 (81.7)	416 (69.3)	746 (90.6)
		Yes	216 (15.2)	171 (28.5)	45 (5.5)
		Missing	45 (3.2)	13 (2.2)	32 (3.9)
	**Enough food to eat**				
		No	1198 (84.2)	439 (73.2)	759 (92.2)
		Yes	185 (13.0)	150 (25.0)	35 (4.3)
		Missing	40 (2.8)	11 (1.8)	29 (3.5)
	**Clean water to drink**				
		No	1228 (86.3)	455 (75.8)	773 (93.9)
		Yes	159 (11.2)	136 (22.7)	23 (2.8)
		Missing	36 (2.5)	9 (1.5)	27 (3.3)
	**Getting needed medicine**				
		No	1163 (81.7)	422 (70.3)	741 (90.0)
		Yes	212 (14.9)	162 (27.0)	50 (6.1)
		Missing	48 (3.4)	16 (2.7)	32 (3.9)
	**Transportation**				
		No	1165 (81.9)	418 (69.7)	747 (90.8)
		Yes	206 (14.5)	165 (27.5)	41 (5.0)
		Missing	52 (3.7)	17 (2.8)	35 (4.3)
**Barriers to testing**					
	No		519 (36.5)	195 (32.5)	324 (39.4)
	Yes		460 (32.3)	203 (33.8)	257 (31.2)
	Missing		444 (31.2)	202 (33.7)	242 (29.4)

### Statistical Analysis

In the parsimonious model, backward selection with Akaike information criterion dropped the following variables: essential worker, vaccinated, and access to health care. Among persons at the time of first testing, all age groups, races, sex at birth, barriers to testing, enough food to eat, clean water to drink, and the DCI and clean water to drink interaction were not statistically associated with the odds of seeing an individual who was symptomatic at a testing location (all *P* values >.05). Individuals with a physical health condition and challenges related to a place to stay/live were statistically more likely to seek testing while being symptomatic, and mental health condition and the DCI and getting needed medicine interaction were moderately so. Those reporting physical health conditions were 85% more likely to have reported being symptomatic (aOR 1.85, 95% CI 1.3-2.65), and those reporting challenges of having a place to stay/live were 307.13 times more likely to have reported being symptomatic (aOR 307.13, 95% CI 1.46-106,372). Those reporting mental health conditions were 53% more likely to have reported being symptomatic (aOR 1.53, 95% CI 0.96-2.48), and those living in a high DCI zip code while also not getting needed medicine were 6% more likely to have reported being symptomatic (aOR 1.06, 95% CI 1.00-1.13). Individuals with a challenge getting needed medicine and transportation as well as the DCI and challenges in having a place to stay/live interaction were statistically less likely to seek testing while symptomatic, and living in a high DCI zip code was moderately so. Participants who had challenges in getting needed medication were 99% less likely to report being symptomatic (aOR 0.01, 95% CI 0.00-0.82). Those who had challenges with transportation were 77% less likely to report being symptomatic (aOR 0.23, 95% CI 0.05-0.64). Those living in a high DCI zip code and facing challenges of having a place to stay/live were 7% less likely to report being symptomatic (aOR 0.93, 95% CI 0.87-0.99), and those living in a high DCI zip code were 1% less likely to seek testing as a symptomatic individual (aOR 0.99, 95% CI 0.98-1.00). Complete results for the logistic regression are displayed in Table 2.

**Table 2.** Adjusted odds ratios and corresponding 95% CIs for logistic regression models. Full discussion of results can be found in the Results: Statistical Analysis section. The parsimonious model was derived using backward selection from the original model. A full description of the procedure can be found in the Methods: Statistical Analysis section.

Variable			Original model		Parsimonious model	
			Adjusted odds ratio	95% CI	Adjusted odds ratio	95% CI
**Age (years)**						
	≤18		1.6	0.9-2.9	1.64	0.94-2.92
	19-29 (reference)		1	—^a^	1	—
	30-39		0.83	0.51-1.37	0.85	0.52-1.4
	40-49		0.97	0.57-1.67	0.98	0.58-1.68
	50-59		0.75	0.42-1.33	0.73	0.42-1.3
	≥60		0.72	0.44-1.17	0.69	0.43-1.1
**Race**						
	White (reference)		1	—	1	—
	Black/African American		0.67	0.39-1.16	0.68	0.4-1.17
	Other		0.7	0.37-1.37	0.71	0.37-1.37
**Sex at birth**						
	Female (reference)		1	—	1	—
	Male		0.87	0.64-1.17	0.87	0.65-1.17
**Essential worker**						
	No (reference)		1	—	1	—
	Yes		1.1	0.76-1.59	—	—
**Vaccinated**						
	No (reference)		1	—	1	—
	Yes		0.88	0.61-1.27	—	—
**Physical health condition**						
	No (reference)		1	—	1	—
	Yes		1.87	1.31-2.68	1.85	1.3-2.65
**Mental health condition**						
	No (reference)		1	—	1	—
	Yes		1.59	1-2.6	1.53	0.96-2.48
**Challenges to health**						
	**Access to health care**					
		No (reference)	1	—	1	—
		Yes	0.52	0-1510	—	—
	**Place to stay/live**					
		No (reference)	1	—	1	—
		Yes	142	0.08-1,151,363	307.13	1.46-106,372
	**Enough food to eat**					
		No (reference)	1	—	1	—
		Yes	0.01	0-1793	2.71	0.79-10.27
	**Clean water to drink**					
		No (reference)	1	—	1	—
		Yes	41	0.06-71,082	66	0.12-112,368
	**Getting needed medicine**					
		No (reference)	1	—	1	—
		Yes	0.01	0-56.8	0.01	0-0.82
	**Transportation**					
		No (reference)	1	—	1	—
		Yes	321	0.01-22,493,690	0.23	0.08-0.64
**Barriers to testing**						
	No (reference)		1	—	1	—
	Yes		1.31	0.95-1.8	1.29	0.94-1.77
**Distress score**			0.99	0.98-1	0.99	0.98-1

a Not applicable.

### Geospatial Analysis

**Figure 1.**
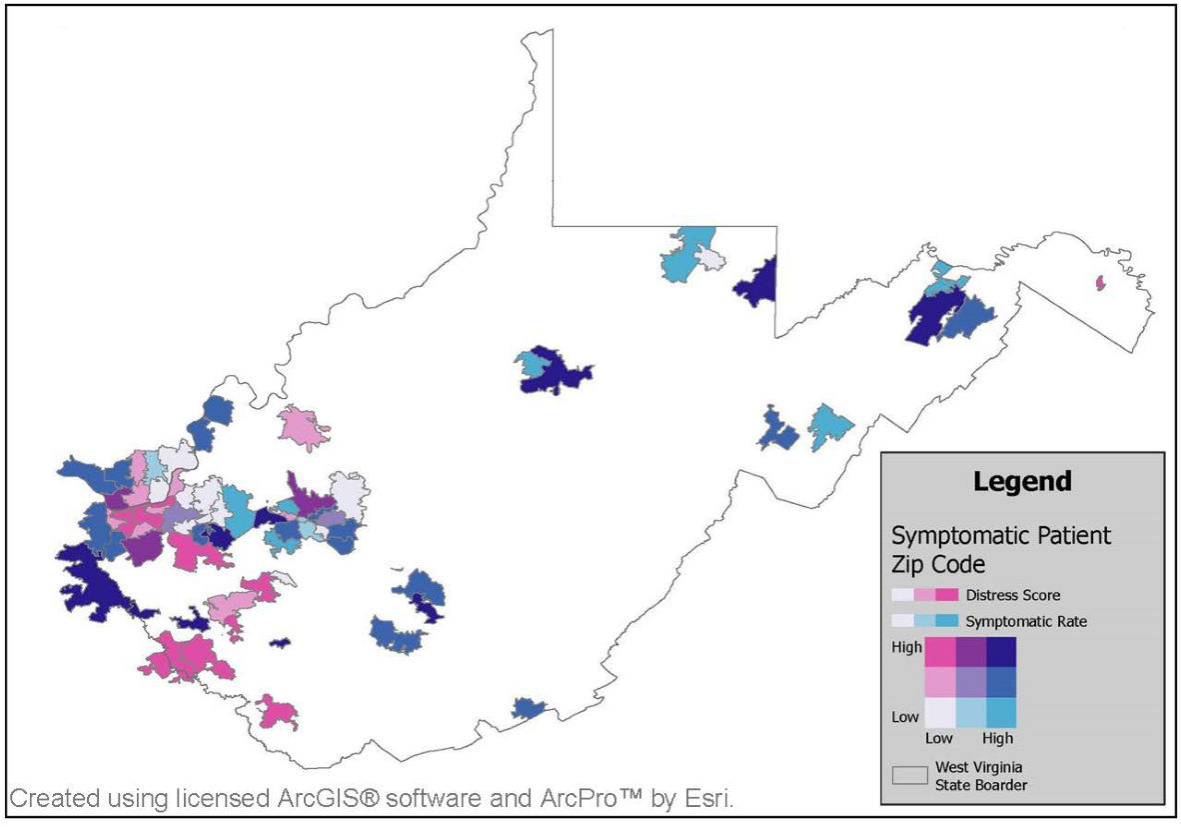
Rate of individuals who were symptomatic per 10 individuals seeking COVID-19 testing at a Rapid Acceleration of Diagnostics for Underserved Populations phase 2 program testing site between May 7 and November 14, 2022, in West Virginia by zip code.

A bivariate map of the zip code–level rate of individuals who were symptomatic per 10 individuals seeking COVID-19 testing and the DCI is displayed in Figure 1. Visually, there appear to be overlapping trends in the DCI and rate of people who are symptomatic per 10 people served at testing locations in the southern and northern regions of West Virginia. In particular, southern West Virginia had more zip codes where the rate of tested people who were symptomatic was low and the DCI was low, indicating fewer people who were symptomatic from nondistressed communities when compared to the rest of the state. There was only 1 zip code in the northern region of West Virginia that followed this trend. However, both regions had zip codes where the rate of tested people who were symptomatic per 10 individuals was high and the DCI score was high, indicating a high number of people who were symptomatic coming from distressed communities. In the southern region, there were many zip codes with a high DCI score but a low rate of people who were symptomatic. This visual observation supports findings from the logistic regression that the DCI was statistically associated with a lower rate of tested people who were symptomatic, particularly for persons in southern West Virginia. When assessing spatial autocorrelation, global Moran *I* did not detect any statistically significant clustering in the rate of people who were symptomatic per 10 individuals tested throughout the RADx-UP study area. Statistically significant clustering was evaluated incrementally across distance thresholds of varying diameters (smallest: 357 km, Moran *I*=0.002, *P*=.63; largest: 784 km, Moran *I*=0.001, *P*=.06) without indication of statistical significance.

## Discussion

### Principal Results

This study identified several factors associated with test seeking among individuals who were symptomatic at RADx-UP2 COVID-19 testing locations. Our study found that individuals with a physical or mental health condition, those facing a challenge in having a place to stay/live, or those with the interaction of living in a high DCI zip code while also not getting needed medicine were more likely to appear at a testing location with symptoms. Additionally, individuals from less distressed communities, who were able to access needed medicine or transportation, and those with the interaction of living in a high DCI zip code and facing challenges in having a place to stay/live were less likely to be symptomatic at the time of their testing. Importantly, we found no statistically significant geographic pattern in the rate of people who were symptomatic per 10 individuals tested. This could suggest that differences observed for persons less likely to be symptomatic by a higher DCI were not due to geographic contexts, such as urban or rural, and perhaps more related to social determinants of health such as facing a challenge in having a place to stay/live within the individual’s zip code of residence. Importantly, these findings address a gap in the existing literature, particularly among studies that utilize recent testing data within epidemiological investigations by looking at underserved areas and the reasoning behind individuals seeking testing [15,17]. Recent testing data reflect a shift toward symptomatic populations who are more likely to struggle with a stable living situation and experience multiple physical or mental health conditions. This is an important consideration, as new information from this study provides an idea of the extent to which the generalizability of testing data is restricted to vulnerable populations or those separate from the general population.

### Physical and Mental Health Conditions

Physical and mental health conditions were found to be associated with individuals presenting with symptoms for COVID-19 testing. Physical health conditions, such as autoimmune disease, hypertension, diabetes, chronic kidney disease, and cardiovascular disease, can cause impediments to the immune system and leave individuals more susceptible to severe illnesses, including COVID-19 [29,30]. These individuals may be more willing to seek out testing when they become symptomatic due to their increased risk of serious illness [4,15,27,29,30]. Altogether these individuals would be more likely to be symptomatic when reporting to testing facilities, whether due to the increased risk of the physical conditions themselves or as a preventative measure taken by the individuals. Additionally, mental health conditions, such as alcohol and substance use, can also increase an individual’s susceptibility to infection from and exposure to COVID-19 [22,31,32]. Mental health conditions can lead to impediments in the immune system, which make an individual more susceptible to COVID-19 and increase situations of greater exposure to COVID-19 [22,31,32]. This is particularly relevant to individuals experiencing homelessness, who are a vulnerable population at high risk for mental health conditions and must undergo COVID-19 testing to gain entrance to shelters [11,15,31-33].

### Challenges to Health and Economic Distress

Those individuals who have challenges in having a place to stay/live and those with the interaction of DCI and getting needed medication were more likely to be symptomatic at the time of testing. These socioeconomic issues could be associated with these individuals being more vulnerable to exposures, leading to more chances of respiratory disease spread due to related aspects such as homelessness or not being able to afford health care such as medication. It was found that individuals who reported challenges in getting needed medicine or transportation, those who lived in distressed communities, and those with the interaction of living in a distressed community while having challenges in having a place to stay/live were less likely to be symptomatic at the time of testing. These associations with not having issues of getting needed medicine or transportation challenges to health could indicate there are fewer travel/access obstacles to the health of an individual as well as fewer people experiencing homelessness in these socioeconomic groups. This could indicate that individuals who are not impeded by these socioeconomic drivers are more likely to seek testing when becoming symptomatic. Coinciding with having no challenges to health, living in higher areas of greater economic distress was associated with lower odds of being symptomatic (Figure 1: pink areas). These findings are interesting because these components measure socioeconomic challenges at both the individual and community levels. These findings give insight into the behaviors of underserved communities that exist across West Virginia when compared to previous studies that look at population-level data and collection methods that would otherwise limit these underserved communities [15,17].

### Limitations

This study has several limitations. First, data for the study comes from questionnaires that are self-reported by the individuals. Due to recall bias or social desirability bias, individuals may be misclassified according to symptomatic status or the presence of a potential predictor [34,35]. Next, many of these symptoms that individuals reported could also be present in the transmission of other pathogens [36]. However, we believe that this did not impact the validity of the study, as the goal was to better understand which factors were associated with the use of testing services in any individual who was symptomatic. Third, individuals who were symptomatic faced challenges to health, such as getting to a testing site or not knowing about available testing, and may not have sought testing. Fourth, the sample size does not indicate confirmed COVID-19 cases—only those who were symptomatic and seeking COVID-19 testing. Finally, the study population is only a subset of the total underserved areas of West Virginia, and some study variables had small sample representation or missing data.

### Conclusions

Overall, this study of symptomatic factors associated with COVID-19 testing in West Virginia emphasized the urgent need to better understand barriers to testing. Despite limitations, this research addresses gaps in the current COVID-19 testing research. This is especially important in underserved areas experiencing disparities, such as the southwestern part of West Virginia (Figure 1). Critical to future public health policy creation is determining why individuals who are symptomatic in high-distress areas are less likely to seek free COVID-19 testing. While factors such as a lack of transportation are possible, there may be other reasons such as belief in the presence of ongoing SARS-CoV-2 transmission or belief in effective prevention (eg, vaccines or quarantine) or treatment.

## Abbreviations

aOR: adjusted odds ratio
CDE: common data element
DCI: Distressed Communities Index
RADx-UP: Rapid Acceleration of Diagnostics for Underserved Populations
SOAR: Solutions Oriented Addiction Response
